# A New Light on Vitamin D in Obesity: A Novel Association with Trimethylamine-N-Oxide (TMAO)

**DOI:** 10.3390/nu11061310

**Published:** 2019-06-10

**Authors:** Luigi Barrea, Giovanna Muscogiuri, Giuseppe Annunziata, Daniela Laudisio, Giulia de Alteriis, Gian Carlo Tenore, Annamaria Colao, Silvia Savastano

**Affiliations:** 1 Dipartimento di Medicina Clinica e Chirurgia, Unit of Endocrinology, Federico II University Medical School of Naples, 80131 Naples, Italy; giovanna.muscogiuri@gmail.com (G.M.); daniela.laudisio@libero.it (D.L.); dealteriisgiulia@gmail.com (G.d.A.); colao@unina.it (A.C.); sisavast@unina.it (S.S.); 2 Department of Pharmacy, University of Naples “Federico II”, 80131 Naples, Italy; giuseppe.annunziata@unina.it (G.A.); giancarlo.tenore@unina.it (G.C.T.)

**Keywords:** vitamin D, obesity, Trimethylamine N-oxide (TMAO), fatty liver index (FLI)

## Abstract

Vitamin D deficiency and obesity are two public health problems extensively exacerbated over the last years. Among the several mechanisms proposed to account for the complex interplay between vitamin D and obesity, one that has gained particular attention is related to the emerging role of obesity-related changes in gut microbiota and gut-derived metabolites, such as Trimethylamine-N-oxide (TMAO). Vitamin D deficiency and high circulating TMAO levels are associated with body weight and the severity of non-alcoholic fatty liver disease (NAFLD). Considering the link of obesity with vitamin D on the one hand and obesity with TMAO on the other hand, and the central role of the liver in both the vitamin D and TMAO metabolism, the aim of this cross-sectional observational study was first, to confirm the possible inverse association between vitamin D and TMAO across different body mass index (BMI) classes and second, to investigate if this association could be influenced by the presence of NAFLD. One hundred and four adult subjects (50 males and 54 females; 35.38 ± 7.49 years) were enrolled. The fatty liver index (FLI) was used as a proxy for the diagnosis of NAFLD. Vitamin D deficiency was found in 65 participants (62.5%), while 33 subjects (31.7%) had insufficient levels, and the remaining subjects had sufficient levels of vitamin D. Subjects with both vitamin D deficiency and FLI-NAFLD had the highest TMAO levels (*p* < 0.001). By stratifying the sample population according to the BMI classes, vitamin D levels decreased significantly along with the increase of plasma TMAO concentrations, with the lowest vitamin D levels and highest TMAO, respectively, in class III obesity. Vitamin D levels showed significant opposite associations with circulating levels of TMAO (*r* = −0.588, *p* < 0.001), but this association was no longer significant after the adjustment for FLI values. The highest values of TMAO were significantly associated with the severity of obesity (OR 7.92; *p* < 0.001), deficiency of vitamin D (OR 1.62; *p* < 0.001), and FLI-NAFLD (OR 3.79; *p* < 0.001). The most sensitive and specific cut-off for vitamin D to predict the circulating levels of TMAO was ≤19.83 ng/mL (*p* < 0.001). In conclusion, our study suggests that high TMAO levels are associated with vitamin D deficiency and NAFLD. Further studies are required to investigate if there is a causality link or whether all of them are simply the consequence of obesity.

## 1. Introduction

Modern and westernised lifestyles, including smoking habits, physical inactivity, and an unhealthy diet are considered one of the major culprits of the association between low vitamin D status and obesity [1]. Low sun exposure due to less outdoor activity and a sedentary lifestyle, vitamin D sequestration, or volumetric dilution in fat mass are some of the factors playing a major role in the pathogenesis of the hypovitaminosis D in patients with obesity [2,3]. Among the several mechanisms proposed to account for the complex interplay between obesity and vitamin D [2,3], one that has gained particular attention is related to the emerging role of obesity-related changes in gut microbiota and gut-derived metabolites, such as Trimethylamine-N-oxide (TMAO) [4,5]. 

TMAO is a gut microbe-generated metabolite developed by a host’s flavin monooxygenase 3 (FMO3) in the liver, with an important role as a predictor of cardiovascular disease risk [6,7]. In particular, TMAO has been shown to exert a pro-atherogenic effect by affecting the metabolism of cholesterol at different steps [5]. Skin 7-dehydrocholesterol is also a well-known precursor of vitamin D [5]. A recent study reported a novel link between vitamin D and plasma TMAO concentrations by showing that vitamin D supplementation at 12 months lowered plasma fasting TMAO levels [8]. Although the composite effect of age, sex, and creatinine was evaluated in this study, the possible confounding effect of body weight and liver fat content was not considered. Of interest, there is evidence that TMAO correlated positively with body mass index (BMI) [9], and that its levels increased along with the classes of BMI [10]. In addition, TMAO has been reported to cause liver inflammation and damage [11], and an adverse association between the circulating TMAO levels and the presence and severity of non-alcoholic fatty liver disease (NAFLD) has also been reported [10,12]. 

Fatty liver index (FLI) is an accurate predictor of NAFLD which incorporates simple anthropometric measurements; in particular, BMI and waist circumference (WC) and metabolic parameters, including triglycerides and liver function [13], are considered a screening tool to identify NAFLD in subjects with insulin resistance and cardio-metabolic risk factors where an ultrasound is unavailable [14,15] and more recently also in lean-NAFLD subjects [16].

Currently, there are no studies available evidencing whether the adverse association between vitamin D and TMAO could be differently affected by BMI and NAFLD. Considering the link of obesity with vitamin D on the one hand and obesity with TMAO on the other hand, and the central role of the liver in both the vitamin D and TMAO metabolism, the aim of this study was to evaluate whether BMI and the presence of NAFLD assessed by FLI could influence the adverse link between vitamin D and TMAO and to determine the cut off value of levels of vitamin D predictive of plasma TMAO concentrations.

## 2. Materials and Methods 

### 2.1. Design and Setting

This was a cross-sectional observational study carried out at the Department of Clinical Medicine and Surgery, Unit of Endocrinology, University Federico II, Naples (Italy), from October 2016 to March 2019. The work was carried out in accordance with the Code of Ethics of the World Medical Association (Declaration of Helsinki) for experiments involving humans, and it was approved by the Ethical Committee of the University of Naples “Federico II” Medical School (n. 173/16). The purpose of the protocol was explained to all the study participants, and written informed consents were obtained. 

### 2.2. Population Study

Recruitment strategies included a sample of 311 adult Caucasians subjects (21–56 years) of both genders consecutively enrolled among patients of our outpatient clinic, hospital volunteers and employees and residing in the Naples metropolitan area (latitude 40°49′ N; elevation 17 m). The subjects were evaluated from March through June 2018. All female subjects were non-pregnant and non-lactating. A full medical history, including drug use, was collected. 

In order to increase the homogeneity of the subject samples, we included only adults of both genders with the following criteria of exclusion:

- Impaired renal function (normal values: Estimated glomerular filtration rate ≥90 mL/min/1.73 m^2^ calculated by a chronic kidney disease epidemiology collaboration equation; Chronic Kidney Disease Epidemiology Collaboration (CKD EPI) (9 subjects);
- Chronic liver diseases, viral hepatitis patients, hemochromatosis, hepatic malignancy (13 subjects);
- Presence of type 2 diabetes (T2DM) (defined by the criteria of the American Diabetes Association as follows: Basal plasma glucose level ≥ 126 mg/dL on two occasions, or glycated haemoglobin (HbA1c) ≥ 6.5% (≥48 mmoL/moL) on two occasions, or both at the same time. Participants on antidiabetic medication were considered to have T2DM [17] (21 subjects);
- Uncontrolled thyroid or parathyroid disease (33 subjects);
- Clinical atherosclerosis (coronary artery disease, peripheral vascular disease) (36 subjects);
- Current therapy with calcium, vitamin D supplementation, or osteoporosis therapies, anti-inflammatory drugs, statin and other hypolipidemic agents (41 subjects);
- User of antibiotics or probiotics within two months of recruitment (15 subjects);
- Specific nutritional regimens, including vegan or vegetarian diets (three subjects);
- Vitamin/mineral or antioxidant supplementation (29 subjects);
- Alcohol abuse according to the Diagnostic and Statistical Manual of Mental Disorders (DSM)-V diagnostic criteria (7 subjects).

**Figure 1:**
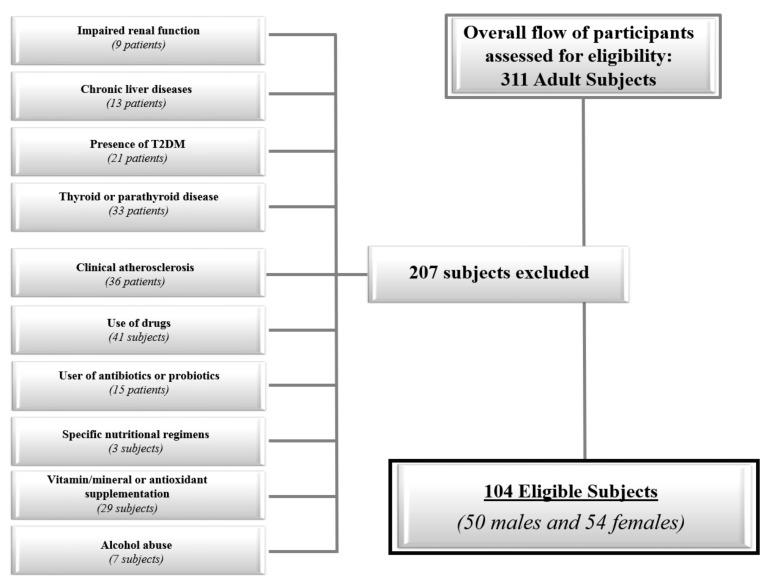
The flow chart of the study subjects.

The flow chart of the studied subjects is shown in Figure 1.

### 2.3. Anthropometric Measurements and Blood Pressure

Measurements were performed between 8 and 12 AM, after an overnight fast. The measurements were made in a standard way by the same operator (a nutritionist experienced in providing nutritional assessment and body composition). At the beginning of the study, all anthropometric measurements were taken with subjects wearing only light clothes and without shoes. For each subject, weight and height were measured to calculate the BMI (weight (kg) divided by height squared (m^2^), kg/m^2^). Height was measured to the nearest 0.5 cm using a wall-mounted stadiometer (Seca 711; Seca, Hamburg, Germany). Body weight was determined to the nearest 0.1 kg using a calibrated balance beam scale (Seca 711; Seca, Hamburg, Germany). BMI was classified according to (World Health Organization) WHO’s criteria with a normal weight being 18.5–24.9 kg/m^2^; overweight, 25.0–29.9 kg/m^2^; grade I obesity, 30.0–34.9 kg/m^2^; grade II obesity, 35.0–39.9 kg/m^2^; and grade III obesity ≥ 40.0 kg/m^2^ [18]. In accordance with the National Center for Health Statistics [19], WC was measured to the closest 0.1 cm using a non-stretchable measuring tape at the natural indentation or at a midway level between the lower edge of the rib cage and iliac crest if no natural indentation was visible, as previously reported [20,21,22,23]. 

### 2.4. Assay Methods

Samples were collected in the morning between 8 and 10 a.m., after an overnight fast of at least 8 h, and stored at −80 °C until processed. All biochemical analyses including fasting plasma glucose, total cholesterol, fasting plasma triglycerides, Alanine Transaminase (ALT), Aspartate Aminotransferase (AST), and γ-Glutamyltransferase (γGT) were performed with a Roche Modular Analytics System in the Central Biochemistry Laboratory of Federico II University Hospital (Naples, Italy). Low-density lipoprotein (LDL) cholesterol and high-density lipoprotein HDL cholesterol were determined by a direct method (homogeneous enzymatic assay for the direct quantitative determination of LDL and HDL cholesterol). Fasting insulin levels were measured by a solid-phase chemiluminescent enzyme immunoassay using commercially available kits (Immunolite Diagnostic Products Co, Los Angeles, CA, USA), as previously reported in several studies [24,25,26,27]. Serum levels of vitamin D (25-OH vitamin D) were measured with chemiluminescence (Liaison, DiaSorin, Saluggia, Italy). Vitamin D deficiency was defined as a serum concentration of vitamin D < 20 ng/mL (50 nmoL/L), with insufficiency found in levels between 21 and 29 ng/mL (from 52.5 to 72.5 nmoL/L), and normal levels being defined as values of vitamin D ≥ 30 ng/mL (75 nmoL/L) [28]. TMAO serum levels were measured in samples stored at −80 °C. A previous study indicated that, under these conditions, TMAO is stable for several years [29]. The quantification of plasma TMAO concentrations was performed using the method described by Beale and Airs [30] and reported in our previous study [10,31]. Briefly, serum proteins were precipitated with methanol (serum:methanol, 1:2, *v*/*v*); samples were vortex-mixed for 2 min, centrifuged at 14,000 g for 10 min (4 °C) [32], and supernatants were collected and analyzed by the High Performance Liquid Chromatography–Mass Spectrometry (HPLC/MS) method. Both HPLC/MS conditions and method optimization were performed in accordance with Beale and Airs [30]. The HPLC system Jasco Extrema LC-4000 system (Jasco Inc., Easton, MD, USA) was coupled to a single quadrupole mass spectrometer (Advion ExpressIonL CMS, Advion Inc., Ithaca, NY, USA) equipped with an Electrospray ionization (ESI) source, operating in positive ion mode. Chromatographic separation was performed with a Luna HILIC column (150 × 3 mm, 5 µm particles) in combination with a guard column (HILIC), both supplied by Phenomenex (Torrance, CA, USA). The intra-assay coefficients of variations (CV) was <5.5%, as already widely reported in our previous studies [32,33,34,35,36]. FLI was calculated with the formula: (FLI = eL/(1 + eL) × 100, L = 0.953 × loge triglycerides + 0.139 BMI + 0.718 × logeγGT + 0.053 × WC−15.745). The value of FLI that was considered as the cut-off value on the basis of Bedogni’s criterion to detect the presence of NAFLD was 30 [13].

### 2.5. Statistical Analysis

Data distribution was evaluated by a Kolmogorov–Smirnov test and the abnormal data were normalized by a logarithm. Skewed variables (BMI, TMAO, vitamin D, fasting glucose, insulin, total cholesterol, HDL cholesterol, LDL cholesterol, triglycerides, ALT, AST, γGT, and FLI) were back-transformed for presentation in tables and figures. Results are expressed as mean ± standard deviation (SD). Differences according to the absence/presence of FLI-NAFLD were analyzed by a Student’s unpaired *t*-test. The differences among the vitamin D categories and classes of BMI were analyzed by ANOVA followed by the Bonferroni post-hoc test. The correlations between study variables were performed using Pearson *r* and correlation coefficients were estimated after adjusting for FLI. Proportional Odds Ratio (OR) models, 95% Interval Confidence (IC), and R^2^, were performed to assess the association among classes of BMI, vitamin D categories, and absence/presence of FLI-NAFLD. Receiver operator characteristic (ROC) curve analysis was performed to determine sensitivity and specificity, the area under the curve (AUC), and IC, as well as cut-off values of levels of vitamin D in detecting circulating levels of TMAO. The test AUC for ROC analysis was also performed. To avoid multicollinearity, variables with a variance inflation factor (VIF) > 10 were excluded. Values ≤0.05% were considered statistically significant. Data were stored and analyzed using the MedCalc^®^ package (Version 12.3.0 1993–2012 MedCalc Software bvba—MedCalc Software, Mariakerke, Belgium). 

## 3. Results

The study population consisted of 104 participants, 50 males and 54 females. The anthropometric characteristics, systolic blood pressure (SBP) and diastolic blood pressure (DBP), plasma TMAO, vitamin D, and metabolic profile of the study population are reported in Table 1. Most of the participants had grade III obesity (27.9%) and vitamin D deficiency (62.5%). FLI-NAFLD was present in more than half of the participants (59.6%). 

**Figure 2:**
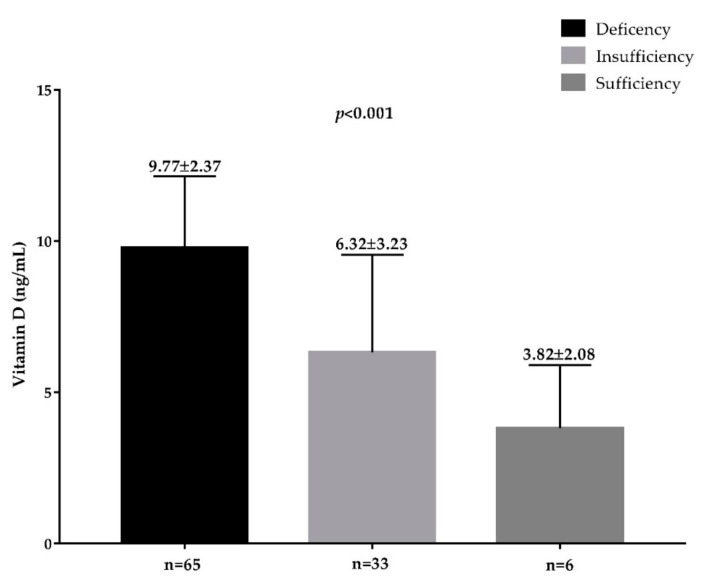
The circulating levels of TMAO in the population study across vitamin D categories. A *p* value in bold denotes a significant difference (*p* < 0.05); *n* = number.

**Figure 3:**
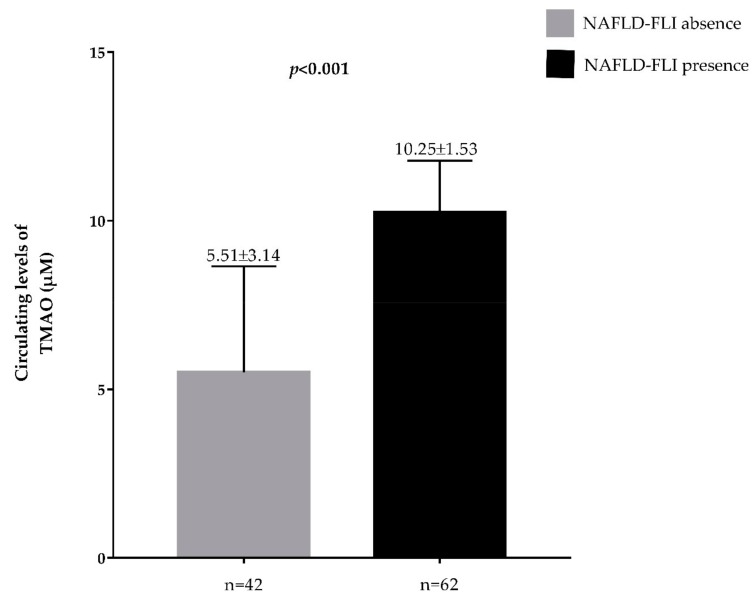
Circulating levels of TMAO in the population study classified according to presence/absence of FLI-NAFLD. A *p* value in bold denotes a significant difference (*p* < 0.05); *n* = number.

Plasma TMAO concentrations across vitamin D categories and according to presence/absence of FLI-NAFLD are reported in Figure 2 and Figure 3, respectively. The highest plasma TMAO concentrations were present in the subjects with a vitamin D deficiency and FLI-NAFLD, *p* < 0.001 (Figure 2 and Figure 3, respectively). 

Table 2 shows the anthropometric characteristics and metabolic profile of the population grouped on the base of BMI categories. As reported in Table 2, no differences were observed in age (*p* = 0.063), while compared with normal-weight subjects, subjects who were overweight and those with obesity exhibited statistical differences in all study parameters (*p* < 0.001). Stratifying the sample population according to the BMI classes, vitamin D decreased significantly along with the increase of circulating levels of TMAO, with the lowest vitamin D levels and highest TMAO, respectively, in class III obesity.

### Correlation Analysis

**Figure 4:**
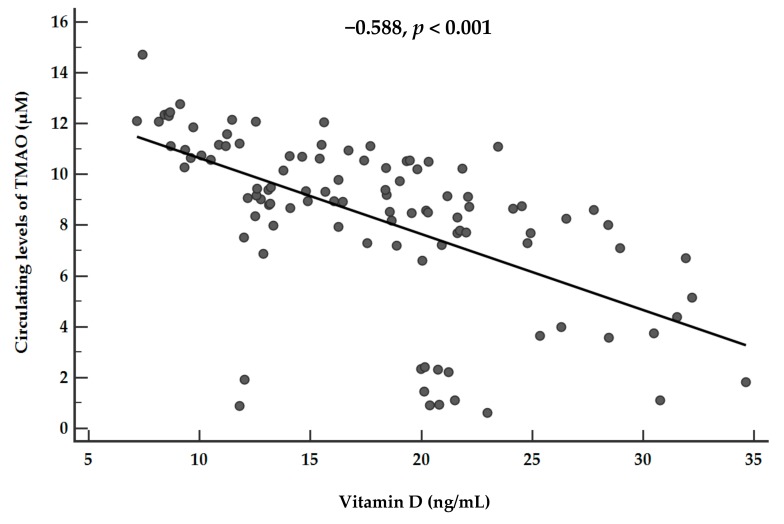
Correlations between plasma vitamin D levels and plasma TMAO concentrations. A *p* value in bold denotes a significant difference (*p* < 0.05).

The correlations of vitamin D and TMAO levels with age, anthropometric measurements, and metabolic profile are summarized in Table 3. Apart from age, vitamin D and circulating levels of TMAO showed significant opposite associations with all parameters. Figure 4 shows the correlation between vitamin D levels and plasma TMAO concentrations (*r* = −0.588, *p* < 0.001). The association was no longer evident after adjusting for FLI-NAFLD (*r* = −0.123, *p*= 0.215).

The results of the bivariate proportional OR model performed to assess the association of TMAO with quantitative variables are reported in Table 4. The highest values of TMAO were significantly associated with the severity of obesity (OR 7.92; *p* < 0.001), deficiency of vitamin D (OR 1.62; *p* < 0.001), and FLI-NAFLD (OR 3.79; *p* < 0.001). 

**Figure 5:**
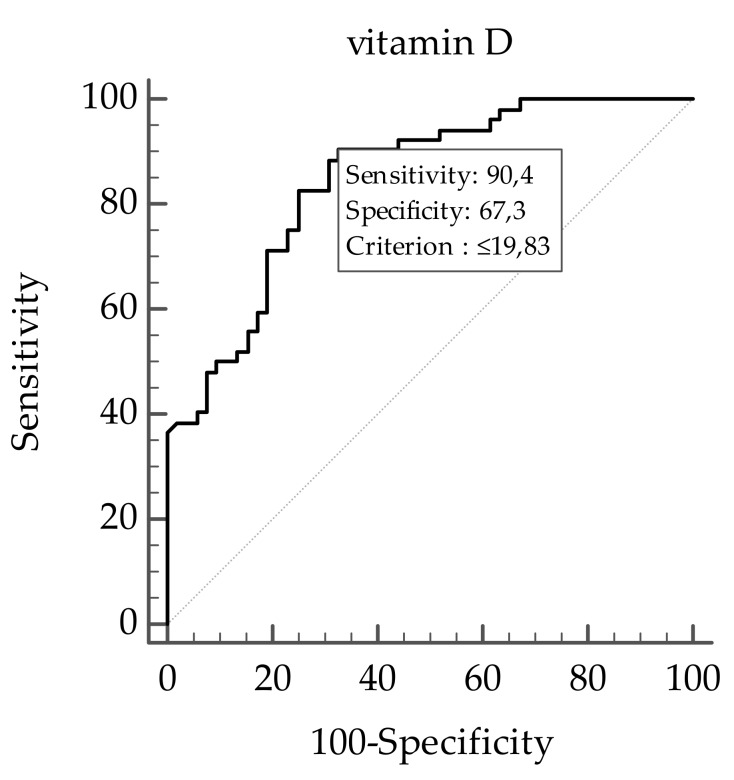
The receiver operator characteristic (ROC) for predictive values of levels of vitamin D in detecting plasma TMAO concentrations. A *p* value in bold denotes a significant difference (*p* < 0.05).

A ROC analysis was then performed to determine the cut off value of levels of vitamin D predictive of plasma TMAO concentrations. In particular, levels of vitamin D ≤ 19.83 ng/mL (*p* < 0.001, AUC 0.846, standard error 0.037, 95% CI 0.762 to 0.909; Figure 5) could serve as a threshold for significantly increased plasma TMAO concentrations above the median (8.95 µM).

## 4. Discussion

In this cross-sectional, observational study, we reported an inverse association between vitamin D and plasma TMAO concentrations in a sample of an adult population stratified according to the categories of BMI and FLI-NAFLD. To the best of our knowledge, this has been the first study to report a negative association between vitamin D and plasma TMAO concentrations in subjects with obesity.

When considering the entire sample population, higher plasma levels of TMAO were observed in study participants with a vitamin D deficiency and in those with presence of FLI-NAFLD. The stratification of the study population according to the BMI classes allowed us to confirm the negative relationship between vitamin D and plasma TMAO concentrations. However, we extended the knowledge on the relationship between vitamin D and TMAO as we observed that this negative association increased along with the BMI; the highest plasma TMAO concentrations were associated with the lowest levels of vitamin D in study participants with class III BMI. In addition, higher plasma TMAO concentrations were significantly associated not only with the severity of obesity and vitamin D deficiency, but also with the presence of FLI-NAFLD. Of interest, the negative association between vitamin D and TMAO was no longer evident after adjusting for FLI-NAFLD. Finally, using ROC curve analysis, vitamin D levels ≤ 19.83 ng/mL predicted the highest plasma TMAO concentrations. 

The negative association between vitamin D and TMAO was actively linked to obesity and obesity-related disorders in an opposite way, which suggests that some considerations need to be taken into account. Epidemiologic studies have reported an association between vitamin D deficiency with cardiovascular risk factors and adverse cardiovascular outcomes [33]. In a meta-analysis of prospective cohort studies based on a large sample population [34], adjusted risk of cardiovascular mortality was 57% higher in the lowest category than in the highest category of vitamin D levels [35]. However, albeit vitamin D has been suggested as a protective cardiovascular disease risk factor [36,37], the scientific evidence still remains inconclusive [38]. In this complex interplay, it could be hypothesized that vitamin D deficiency could have a direct role in determining the increased risk of developing cardiovascular diseases but also that vitamin D deficiency and cardiovascular risk could be both determined by obesity [39,40]. However, it cannot be overlooked that vitamin D deficiency may contribute to worsening cardiovascular health in obesity through several direct mechanisms, including activation of the renin-angiotensin-aldosterone system, which can predispose obese individuals to hypertension and left ventricular hypertrophy or an increase in the parathyroid hormone, which increases insulin resistance, downregulation proinflammatory cytokines and metalloproteinases, upregulation of anti-inflammatory cytokines, and inhibitors of metalloproteinases [41,42]. The opposite association of vitamin D levels and plasma TMAO concentrations across BMI classes and the presence of FLI-NAFLD allowed us to speculate that the increased plasma TMAO concentrations could be a further mechanism through which vitamin D deficiency could contribute to worsening cardiovascular risk in subjects with obesity. 

Plasma TMAO concentrations increase after the gut microbial metabolism of high-fat food found in the Western Diet, including red meat, eggs, and dairy products, which are rich in L-carnitine and choline. The metabolic pathway of TMAO includes the digestion of these amines from gut microbiota with the production of trimethylamine, which is then converted to TMAO via FMO3 in the liver [43,44]. Randrianarisoa et al. [9] reported that TMAO correlated positively with BMI, insulin resistance, visceral fat mass, and liver fat content [9]. Very recently, we reported a novel association between circulating levels of TMAO and the Mediterranean diet in healthy normal-weight adults, with a clear gender difference in this association [31], and FLI [10]. 

A large body of evidence supports the association of TMAO with increased cardiovascular risk. A recent meta-analysis of 11 prospective cohort studies indicated that plasma TMAO correlated with a 23% increase in risk for cardiovascular events ((hazard ratio) HR 1.23, 95% CI 1.07 to 1.42) and a 55% increase in all-cause mortality [45]. Given the association between TMAO and cardiovascular risk and the tight link between gut microbiota and obesity/obesity-related diseases, it has been hypothesized that the TMAO pathway may also be mechanistically linked to the pathogenesis of obesity [46]. 

Obeid R et al. reported that a 12-month supplementation of vitamin D was effective in lowering plasma fasting TMAO and increasing plasma choline, a TMAO precursor and basic component for the synthesis of phosphatidylcholine, which in turn is necessary for promoting lipid exportation from the liver [8]. The authors suggested that their findings were the result of the vitamin D effect on the metabolism of choline, a dietary nutrient whose deficiency is linked to the accumulation of hepatic lipid [47]. As a further linking mechanism, obesity-related vitamin D deficiency could blunt gut homeostasis, i.e., the regulation of the integrity of the intestinal epithelium and the mucosal immune system along with the microbial communities, thus altering TMAO pathway [48,49]. 

On the other hand, TMAO and TMAO-producing enzyme FMO3 in the liver affect the metabolism of cholesterol by altering the enterohepatic cholesterol and bile acid metabolism [5]. Thus, it is conceivable that TMAO could also affect the metabolism of 7-dehydrocholesterol, the skin precursor of vitamin D, lending support to the hypothesis that the interactions between vitamin D and TMAO could be bidirectional.

The results of this study confirmed, however, that the presence of FLI-NAFLD was significantly and positively associated with plasma TMAO concentrations, while also highlighting that the inverse association between vitamin D levels and plasma TMAO concentrations was no longer significant after adjusting for FLI-NAFLD presence. The liver has an important role in the metabolism of both vitamin D and TMAO through 25-hydroxyvitamin D-hydroxylase and the TMAO-producing enzyme FMO3, respectively. On the other hand, vitamin D deficiency is involved in an increased hepatic lipogenesis and a reduced beta-oxidation, which leads to the accumulation of fat in the liver, accompanied by insulin resistance [50]; however, no direct associations between vitamin D deficiency and severity of liver steatosis has been reported in humans [51]. In addition, very recently, it was demonstrated that, in animal models, TMAO impaired liver function and increased hepatic triglyceride accumulation and lipogenesis, while in humans, TMAO worsened liver steatosis by modulating bile acid metabolism, by shifting their hepatic composition toward a farnesoid X receptor-antagonistic activity [52]. Thus, a bidirectional relationship could operate also between TMAO and NAFLD. FLI is a surrogate marker of a fatty liver widely used as a screening tool to identify NAFLD in epidemiological studies [14,15,16]. Of interest, Miao et al. [10,53] found that an animal model characterized by a selective hepatic insulin resistance has increased circulating TMAO levels associated with the up-regulation of the TMAO-producing enzyme FMO3 in the liver. In this context, hepatic FMO3 is a target of insulin action [53] and its activity is boosted in the condition of insulin resistance [54]. Therefore, the link between TMAO and cardiovascular diseases might represent the epiphenomenon of hepatic insulin resistance. However, it has been proposed that TMAO alone could affect hepatic triglycerides levels and alter the synthesis and transport of bile acids [12], while the addition of 0.2% TMAO for four weeks in mice on a high-fat diet impaired glucose tolerance, blocked the hepatic insulin signaling pathway, and caused adipose tissue inflammation [55], thus paving the way to the development of NAFLD, the metabolic disease of the liver associated with insulin resistance. Accordingly, studies in humans have confirmed the positive association of circulating TMAO levels with the presence and severity of NAFLD, both by ultrasonographic NAFLD evaluation [12] and FLI, and also independently of common confounding variables, including BMI [10]. 

The cross-sectional design is the main limitation of this study, as it does not allow the identification of any causal association between low vitamin D status and increased plasma TMAO concentrations. In addition, we did not include an analysis of gut microbiota, nutritional assessment, or evaluation of metabolic precursor of TMAO, such as choline, betaine, or carnitine. However, the study of gut microbiota is burdened by a high intra-individual variability that might affect the interpretation of the results [56,57,58,59]. In addition, the influence of diet on fasting TMAO concentrations, albeit statistically significant, could be considered rather moderate [31,60]. Moreover, although the liver biopsy is the gold-standard technique for identifying NAFLD, we used a surrogate marker of NAFLD which is FLI that has been demonstrated to be tightly correlated to the assessment of NAFLD by an ultrasound. Moreover, a liver biopsy is an invasive procedure burdened with rare but potentially life-threatening complications, whereas FLI has proved to represent an easy screening tool to identify NAFLD in patients with cardio-metabolic risk factors [13,61,62]. Due to the lack of a liver biopsy, the association between vitamin D and TMAO in the setting of NAFLD should be investigated in a future study. Finally, our study was based on a single clinical center, with a possibility of selection bias in the results. Nonetheless, the single-center study allowed us to increase the homogeneity of the sample as we included participants living in the same geographical area, with the same effect of latitude on vitamin D levels and, likely, with a similar nutrient availability and food consumption pattern. Nevertheless, a major strength of this study is the stratification of our sample population across BMI classes, and the inverse association between vitamin D status and plasma TMAO levels in each group was compared and evaluated with that of the other groups. In addition, we carefully excluded from the enrollment those participants who had any of the main known factors that can affect TMAO metabolism, such as impaired renal or liver function, T2DM, and alcohol abuse. 

In conclusion, our study further extends the knowledge on the adverse link between vitamin D and TMAO as a possible adjunctive factor involved in cardiovascular risk in obesity. The results of our study allowed us to speculate that the role of TMAO as a marker of cardiovascular risk could also be mediated through its association with a low vitamin D status or the presence of NAFLD in subjects with obesity. However, the potential translational application of the results of this study into clinical practice requires large-scale data to confirm the inverse association between vitamin D and plasma TMAO concentrations in obesity and obesity-related diseases and to clarify the causality, if any.

## Figures and Tables

**Table 1:** Anthropometric characteristics, blood pressure, plasma Trimethylamine-N-oxide (TMAO), vitamin D, and metabolic profile of the study population.

Parameters	Mean ± SD or Number (%)
Age (years)	35.38 ± 7.49
BMI (kg/m^2^)	33.52 ± 9.59
Normal weight	25, 24.0%
Overweight	23, 22.1%
Grade I obesity	15, 14.4%
Grade II obesity	12, 11.5%
Grade III obesity	29, 27.9%
WC (cm)	109.14 ± 24.34
SBP (mmHg)	124.27 ± 13.06
SDP (mmHg)	79.86 ± 10.46
Plasma TMAO (µM)	8.33 ± 3.28
Vitamin D (ng/mL)	17.69 ± 6.44
Deficiency	65, 62.5%
Insufficiency	33, 31.7%
Normal	6, 5.8%
Fasting Glucose (mg/dL)	100.56 ± 17.48
Insulin (µU/mL)	13.68 ± 12.57
Total cholesterol (mg/dL)	184.65 ± 40.00
HDL cholesterol (mg/dL)	46.50 ± 12.10
LDL cholesterol (mg/dL)	111.51 ± 42.69
Triglycerides (mg/dL)	139.30 ± 54.74
ALT (U/L)	31.96 ± 15.25
AST (U/L)	32.49 ± 15.61
γGT (U/L)	35.71 ± 20.04
FLI	63.78 ± 33.89
FLI NAFLD (presence)	62, 59.6%

A *p* value in bold denotes a significant difference (*p* < 0.05).

**Table 2:** Age, anthropometric measurements, and metabolic profile of participants grouped on the basis of BMI categories.

Parameters	Normal Weight *n* = 25; 24.0%	Over Weight *n* = 23; 22.1%	Grade I Obesity *n* = 15; 14.4%	Grade II Obesity *n* = 12; 11.5%	Grade III Obesity *n* = 29; 27.9%	*p*-Value
Age (years)	32.64 ± 7.55	38.35 ± 7.40	37.60 ± 6.45	35.00 ± 8.68	34.41 ± 6.81	0.063
BMI (kg/m^2^)	23.11 ± 1.59	27.34 ± 1.24	32.37 ± 1.47	37.58 ± 1.43	46.31 ± 5.31	**<0.001**
WC (cm)	84.17 ± 10.66	95.60 ± 12.21	109.41 ± 8.08	117.91 ± 14.33	137.64 ± 16.41	**<0.001**
Plasma TMAO (µM)	3.53 ± 2.49	8.32 ± 0.70	9.17 ± 1.11	9.94 ± 0.90	11.39 ± 1.03	**<0.001**
Vitamin D (ng/mL)	23.69 ± 6.35	20.29 ± 3.94	17.27 ± 4.52	14.78 ± 2.45	11.89 ± 4.11	**<0.001**
Fasting Glucose (mg/dL)	85.60 ± 10.57	95.78 ± 13.15	98.33 ± 13.76	97.00 ± 12.28	119.89 ± 11.15	**<0.001**
Insulin (µU/mL)	2.66 ± 1.15	7.16 ± 5.66	10.64 ± 5.65	14.51 ± 10.77	29.57 ± 9.14	**<0.001**
Total cholesterol (mg/dL)	151.36 ± 20.99	175.61 ± 27.92	168.67 ± 24.38	211.58 ± 43.25	217.66 ± 35.08	**<0.001**
HDL cholesterol (mg/dL)	57.32 ± 8.45	51.43 ± 6.74	41.93 ± 13.38	41.67 ± 10.59	37.62 ± 8.98	**<0.001**
LDL cholesterol (mg/dL)	74.77 ± 23.59	99.33 ± 25.22	99.51 ± 16.81	138.05 ± 46.00	147.67 ± 40.54	**<0.001**
Triglycerides (mg/dL)	96.36 ± 26.22	124.22 ± 21.03	174.47 ± 64.77	159.33 ± 35.22	161.83 ± 65.63	**<0.001**
ALT (U/L)	23.84 ± 6.53	25.08 ± 9.96	36.47 ± 11.30	37.58 ± 17.71	39.76 ± 19.22	**<0.001**
AST (U/L)	21.56 ± 5.95	27.73 ± 6.53	35.70 ± 15.27	39.08 ± 15.44	41.28 ± 19.81	**<0.001**
γGT (U/L)	25.20 ± 7.51	25.65 ± 10.81	38.13 ± 15.93	40.00 ± 14.70	49.72 ± 27.02	**<0.001**
FLI	19.83 ± 13.66	45.02 ± 20.58	78.67 ± 11.27	89.88 ± 7.33	98.04 ± 2.55	**<0.001**
FLI-NAFLD (presence)	0, 0%	7, 30.4%	14, 93.3%	12, 100%	29, 100%	**<0.001**

A *p* value in bold denotes a significant difference (*p* < 0.05).

**Table 3:** Correlations of vitamin D levels and plasma TMAO with age, anthropometric measurements, and metabolic profile.

Parameters	Vitamin D (ng/mL)		TMAO (µM)	
	*r*	*p*-Value	*r*	*p*-Value
Age (years)	−0.055	0.580	0.174	0.078
BMI (kg/m^2^)	−0.752	**<0.001**	0.827	**<0.001**
WC (cm)	−0.631	**<0.001**	0.741	**<0.001**
Fasting Glucose (mg/dL)	−0.561	**<0.001**	0.756	**<0.001**
Insulin (µU/mL)	−0.654	**<0.001**	0.656	**<0.001**
Total cholesterol (mg/dL)	−0.493	**<0.001**	0.631	**<0.001**
HDL cholesterol (mg/dL)	0.482	**<0.001**	−0.676	**<0.001**
LDL cholesterol (mg/dL)	−0.502	**<0.001**	0.653	**<0.001**
Triglycerides (mg/dL)	−0.491	**<0.001**	0.495	**<0.001**
ALT (U/L)	−0.351	**<0.001**	0.417	**<0.001**
AST (U/L)	−0.301	**<0.001**	0.502	**<0.001**
γGT (U/L)	−0.339	**<0.001**	0.462	**<0.001**
FLI	−0.647	**<0.001**	0.826	**<0.001**

A *p* value in bold denotes a significant difference (*p* < 0.05).

**Table 4:** Bivariate proportional odds ratio model to assess the association between circulating levels of TMAO and vitamin D categories and FLI-NAFLD.

Parameters	Circulating Levels of TMAO (µM)			
	OR	*p*-Value	95% IC	R^2^
BMI				
Normal weight	0.08	0.004	0.016–0.465	0.588
Overweight	0.99	0.009	0.866–1.150	0.232
Grade I obesity	0.06	0.004	0.915–1.345	0.012
Grade II obesity	0.02	0.002	0.972–1.650	0.038
Grade III obesity	7.92	<0.001	3.174–19.77	0.525
Vitamin D				
Deficit	1.62	<0.001	1.32–1.99	0.302
Insufficiency	0.75	<0.001	0.65–0.87	0.161
Sufficiency	0.68	0.004	0.53–0.89	0.094
FLI-NAFLD	3.79	<0.001	2.06–6.89	0.518

A *p* value in bold denotes a significant difference (*p* < 0.05).

## Abbreviations

TMAO; Trimethylamine-N-oxide; NAFLD, Non-alcoholic Fatty Liver Disease; BMI, body mass index; FLI, Fatty Liver Index; FMO3, Flavin-monooxygenase-3; WC, Waist Circumference; T2DM, Type 2 Diabetes Mellitus; ALT, Alanine Transaminase; AST, Aspartate Aminotransferase; γGT, γ-Glutamyltransferase; LDL, Low-Density Lipoprotein; HDL, High-density Lipoprotein; SD, Standard Deviation; OR, Odds Ratio; IC, Interval Confidence; ROC, Receiver Operator Characteristic; AUC, Area Under Curve.
